# Spatiotemporal Interaction Residual Networks with Pseudo3D for Video Action Recognition

**DOI:** 10.3390/s20113126

**Published:** 2020-06-01

**Authors:** Jianyu Chen, Jun Kong, Hui Sun, Hui Xu, Xiaoli Liu, Yinghua Lu, Caixia Zheng

**Affiliations:** 1 College of Information Sciences and Technology, Northeast Normal University, Changchun 130117, China; chenjy223@nenu.edu.cn (J.C.); xuh504@nenu.edu.cn (H.X.); 2 Institute for Intelligent Elderly Care, College of Humanities & Sciences of Northeast Normal University, Changchun 130117, China; kongjun@nenu.edu.cn (J.K.); liucz474@nenu.edu.cn (H.S.); 3 Key Laboratory of Applied Statistics of MOE, Northeast Normal University, Changchun 130024, China; 4 Department of Chemical & Biomolecular Engineering, National University of Singapore, Singapore 117585, Singapore; chelxi@nus.edu.sg

**Keywords:** video action recognition, spatiotemporal representation learning, two-branches network, pseudo3D architecture

## Abstract

Action recognition is a significant and challenging topic in the field of sensor and computer vision. Two-stream convolutional neural networks (CNNs) and 3D CNNs are two mainstream deep learning architectures for video action recognition. To combine them into one framework to further improve performance, we proposed a novel deep network, named the spatiotemporal interaction residual network with pseudo3D (STINP). The STINP possesses three advantages. First, the STINP consists of two branches constructed based on residual networks (ResNets) to simultaneously learn the spatial and temporal information of the video. Second, the STINP integrates the pseudo3D block into residual units for building the spatial branch, which ensures that the spatial branch can not only learn the appearance feature of the objects and scene in the video, but also capture the potential interaction information among the consecutive frames. Finally, the STINP adopts a simple but effective multiplication operation to fuse the spatial branch and temporal branch, which guarantees that the learned spatial and temporal representation can interact with each other during the entire process of training the STINP. Experiments were implemented on two classic action recognition datasets, UCF101 and HMDB51. The experimental results show that our proposed STINP can provide better performance for video recognition than other state-of-the-art algorithms.

## 1. Introduction

With the rapid development of mobile phones and digital video recorders, the number of videos has grown explosively. For example, over 300 h of video data are uploaded every minute on YouTube [1]. Due to this explosive growth of videos, people cannot manually process and extract useful information from the video data quickly and accurately. Therefore, how to automatically recognize and analyze the contents of a video has attracted widespread attention in the computer vision community and has rapidly become a significant research topic.

Video action recognition aims to use machine learning techniques to automatically identify human action in the video sequences, which has excellent academic value and broad application prospects, such as in video retrieval [2], intelligent human-machine interfaces [3], intelligent video surveillance [4], and autonomous driving vehicles [5]. However, due to the different motion speeds, pose changes, appearance variations, and camera views of human action in videos, action recognition remains a challenging task [5,6].

The key step in video action recognition is extracting the effective spatiotemporal features where the spatial feature is mainly used to describe the global scene configuration and the appearance of objects in a single frame of the video, while the temporal feature is extracted to represent motion cues among multiple frames over time. In recent years, many video action recognition methods have been proposed, which can be mainly divided into two categories [7]: hand-crafted feature-based action recognition [8,9], and deep learning network-based action recognition [10,11]. Hand-crafted feature-based methods usually detect key spatiotemporal points in the video and then represent these points with local descriptors, while deep learning-based methods utilize multilayers to automatically and progressively extract high-level features from raw input. Compared to hand-crafted feature-based methods, deep learning-based methods can achieve considerably better action recognition performance because they can learn more discriminative representations of videos. Hence, deep learning-based action recognition methods have attracted increasing attention recently.

Deep convolutional neural networks (CNNs) have been widely applied in the field of static image understanding, and they have achieved remarkable results in many practical tasks, e.g., image classification [12], object detection [13], and semantic segmentation [11]. Hence, many researchers have tried to introduce CNNs pretrained on images to directly learn the features from the individual video frames and then fuse the features of all frames into one feature vector as the representation of the video [14,15]. However, learning the features from individual frames for video representation does not fully exploit the temporal information across consecutive frames, which limits the performance of the video analysis tasks, e.g., dynamic scene recognition [16] and action recognition [7,14,15]. To address this limitation, two-stream CNN-based [17] and 3D CNN-based [7,18] deep learning approaches were proposed, and they rapidly became the two mainstream architectures for video action recognition.

Two-stream CNNs [17] capture the appearance and motion information of the video by applying two CNN architectures separately, which can gain good performance for video action recognition and has the merit of high calculation efficiency. However, it integrates the spatial and temporal information by late fusing the softmax predictions of two CNN models, which fails to fully learn intrinsic spatiotemporal features of the video [19]. To mitigate this problem, Feichtenhofer et al. [19] proposed spatiotemporal multiplier networks by adopting a cross-stream residual connection, which can learn more effective spatiotemporal features. Specifically, they introduced the multiplicative motion gating function into residual networks to construct a two-steam architecture that can ensure that the appearance and motion features interact with each other in the process of network learning.

3D CNNs [18,20] utilize 3D convolution filters and 3D pooling operations to capture the spatiotemporal features from the stacked video frame volumes. Some research has shown that 3D convolutions are a superior approach for extracting both spatial and temporal activations of a video. However, executing deep 3D CNNs leads to high computational time and memory demand [21]. Hence, Qiu et al. [7] presented pseudo3D residual networks (P3D ResNets) to simulate 3D convolutions by building the P3D blocks in the residual networks, which is an economical and effective method to replace 3D convolutions.

**Figure 1:**
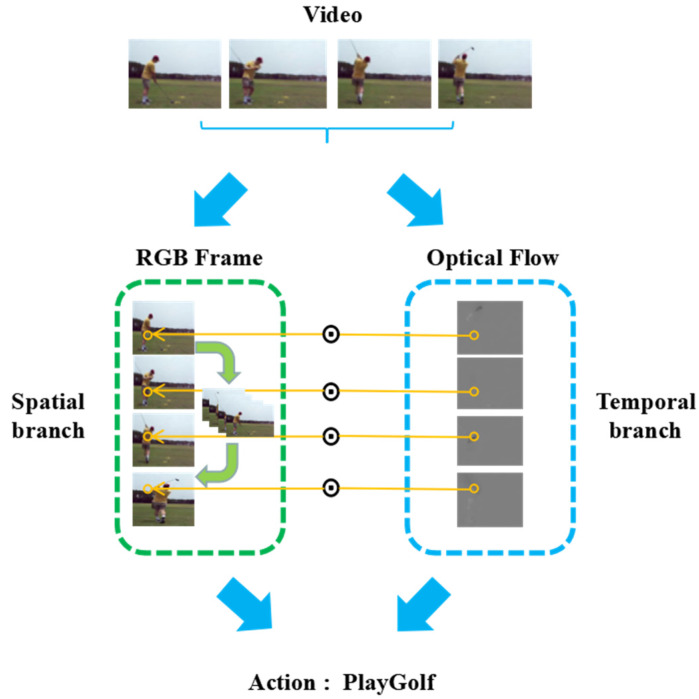
The structure of the spatiotemporal interaction residual network with pseudo3D (STINP). The STINP consists of two branches, the spatial branch and the temporal branch. The spatial branch aims to obtain the features of the scene and objects in the individual frames of the video, where the green arrows represent introducing the pseudo3D structure to extract the interactive relationship among the consecutive frames. The temporal branch employs the optical flow frames as input to obtain the dynamic information of the video.

In this paper, we propose a novel architecture named the spatiotemporal interaction residual network with pseudo3D (STINP) for video action recognition. Figure 1 shows the framework of STINP. The contributions of the STINP are as follows: (1) By introducing a pseudo3D block into two-branch architectures, the proposed STINP can effectively combine the advantages of two-stream and 3D architecture so that it can simultaneously and effectively extract temporal and spatial video information. (2) By employing the multiplicative function to combine the spatial and temporal branches, the STINP can make the learned temporal and spatial representations directly impact each other in the earlier learning stage of the network, and the representations are directly integrated into the final output layer. (3) In the spatial branch of the STINP, 1D temporal convolutions combined with 2D spatial convolutions are added in the residual units by adopting the pseudo3D structure, which aims to learn the interactive cues among the neighboring frames to further improve the effectiveness of the proposed STINP for action recognition tasks.

## 2. Related Work

Video action recognition has been studied for decades, and some progress has been achieved in this field. The earlier studies mainly focused on manually designing an effective feature extraction method to extract and encode the spatiotemporal information of the video. However, in recent years, with the rise of deep learning and large video datasets, an increasing number of studies involve automatically learning the spatiotemporal features of the video via the construction of a deep architecture.

### 2.1. Hand-Crafted Feature-Based Methods

The existing hand-crafted features applied in the area of video action recognition can be generally divided into two categories [22]: global features and local features. Most global feature extraction methods localize and segment human subjects as the region of interest (ROI) and derive the appearance and motion representations from the ROI to form the feature vectors of whole videos. There are many global features designed for action recognition, for example, human skeleton features [23], human contour features [24], and human body shape [25]. In general, due to extracting global features, including the segmentation operation, global features are not robust to occlusions, noise, and perspective changes. Hence, local features are proposed to avoid segmenting the foreground and background of the video by directly extracting the features from the local interest points in the video. The classic local features for action recognition include Harris corners [26], 3D Hessian spatiotemporal interest points [27], and cuboid feature descriptors [28].

In hand-crafted feature-based action recognition methods, the other crucial step is to build a well-performing classifier. The commonly used classification methods are the template-based method [29], the generative model [30], and the discriminative model [31]. The template-based method [8] maps the video action sequences into a set of static patterns and compares the extracted patterns with the previously established templates to estimate the category of the video. The generative model [30,32,33] explicitly learns the actual distribution of each action class by calculating the joint probability distribution and predicts the classes of testing videos by Bayes rules. In contrast to the generative model, the discriminative model [31,34] directly models the conditional probability between the video feature vectors and the action classes, and the testing video is classified as the action class with the highest conditional probability.

### 2.2. Deep Learning Architecture-Based Methods

Because the significant results obtained by CNNs [35] on ImageNet large scale visual recognition challenge(ILSVRC) - [36] demonstrate the strong power of CNNs to learn visual representations, researchers have developed various deep leaning pipelines based on CNNs, making significant breakthroughs [37] in the area of still image classification. Additionally, there have been many attempts to introduce CNNs into the field of video action recognition. Most of these attempts utilize CNNs pretrained on the image datasets to extract the features from every single frame of a video and then fuse the extracted features as a spatiotemporal description of the video using the pooling operation [38], high-dimensional feature encoding [39,40], or recursive neural networks [41]. Karpathy et al. [8] studied different feature fusion strategies, e.g., early fusion, late fusion, and slow fusion, for combining multiple deep-network models to extract the local spatiotemporal representation of large-scale and noisily labeled video datasets. The experimental results show that the accuracy of action recognition is not very satisfactory due to early fusion, late fusion, and slow fusion that do not fully acquire the spatiotemporal information. Hence, to better integrate the spatial and temporal information for action recognition, Simonyan et al. [17] proposed a two-stream convolutional network containing a spatial stream and a temporal stream, in which the temporal stream convolutional network operates on the dense optical flow of multiple-frames, and the spatial stream performs on the still video frames. Tran et al. [42] exploited deep 3D convolutional networks (3D ConvNets) trained on a large-scale video dataset for modeling the appearance and motion simultaneously. Carreira et al. [43] developed a novel two-stream inflated 3D CNN that possesses the advantages and parameters of the 2D CNNs trained on ImageNet to learn the spatiotemporal feature extractors for video. The two-stream and 3D CNN architecture-based convolutional networks have achieved good performance in video recognition tasks because they can simultaneously capture the spatial and temporal cues of the video, however, each has their limitations. For instance, the two-stream-based architectures cannot learn the truly spatiotemporal features because they adopt the late fusing of the separate classification scores of two streams, while the 3D CNN-based approaches have expensive memory demand and computational cost. To avoid the drawbacks of the two-stream and 3D CNN architectures, Feichtenhofer et al. [19] and Qiu et al. [7] separately proposed spatiotemporal multiplier networks and P3D ResNets to recognize the action categories in the video. However, they did not integrate the two-stream and 3D CNN architectures into one framework to further improve the performance of action recognition.

Therefore, we propose a new action recognition model, named the spatiotemporal interaction residual network with pseudo3D (STINP), which can combine the advantage of two-stream and pseudo3D structures to improve action recognition performance.

## 3. Method

The STINP is proposed based on ResNets. In this section, we first review ResNets, and then we give the details of the spatial branch and temporal branch of the STINP, respectively, and the method of integrating these two branches.

### 3.1. Residual Network

In recent years, it has been proven that the depth of the network is a crucial factor for optimizing network performance. In general, a deeper network architecture is beneficial for achieving good image classification task results. Hence, many very deep networks have been designed, such as networks with depths of sixteen [44], twenty-two [45], or thirty [46]. However, as the depth of the network increases, the problems of vanishing and exploding gradient are magnified, and the accuracy of the model degrades rapidly after it reaches the saturated point [12]. To solve the problem of vanishing and exploding gradient, residual networks (ResNets) [12] were proposed.

ResNets employ residual units to learn the residual representation of the input signal. Residual units include the bottleneck building blocks, residual connections, and batch normalization layers. The bottleneck architectures consist of the convolution (1 × 1, 3 × 3, and 1 × 1) layers, and the residual connection adds a skip/shortcut connection to address the vanishing/exploding gradient problem. The residual learning block in ResNets is defined as:

(1) $$X_{l+1}=f(X_{l}+F(X_{l},\{W_{l}\})$$

where $X_{l}$ and $X_{l+1}$ are the input and output data of the l-th residual unit, F is a function that is used to learn the residual map, $W_{l}$ represents the convolution filter, and f represents the rectified linear units (*ReLU*) function.

Although ResNets have obtained good performance for various image classification tasks, they cannot achieve very satisfactory results when they are directly adopted for action recognition in video. This is because ResNets cannot learn the temporal information of the video. Therefore, to simultaneously learn the spatial and temporal cues in the video for action recognition, we propose the STINP, which includes two branches: spatial branch and temporal branch. Both branches are constructed based on ResNets architecture.

### 3.2. Spatial Branch

Generally, the categories of actions are associated with the appearances of objects and scenes appearing in the video [23]. For instance, in subway stations, people often crowd or walk, while in a bookstore, people usually move slowly, stand up, or read a book. Hence, to capture the cues from the objects and the scene in the video and obtain the potential interaction information among the consecutive frames, we designed the spatial branch in the proposed STINP.

Because ResNets can efficiently and effectively extract the features from the images (frames), we utilize it as the underlying architecture of the spatial branch. Specifically, we add the appropriate temporal convolution filters in the “bottleneck” building block of the original ResNets model to enhance the network for capturing not only the appearance features from the single frames but also the interaction features among the adjacent frames. To achieve this, we build our spatial branch by adopting the work in Ref. [7]. That is, we simulate 3D convolutions with 2D convolutional filters in the spatial domain, plus 1D convolutional filters in the temporal domain to obtain the connections on neighbor feature maps. The different combinations of 2D and 1D convolutional filters yield different performances. Hence, we developed two variant structures of the spatial branch in the proposed STINP and we named the STINP with these two different appearance branches as STINP-1 and STINP-2, respectively.

Spatial branch in STINP-1: the 2D convolutional filter (*cf*_2_) and 1D convolutional filter (*cf*_1_) are parallelly combined, which can ensure that both the 2D and 1D convolutional filters directly influence the output of the spatial branch, while they do not directly affect each other. This combination can be expressed as:

(2) $$X_{l+1}=f(X_{l}+cf_{2}(X_{l})+cf_{1}(X_{l}))$$

Spatial branch in STINP-2: the 2D convolutional filter (*cf*_2_) and 1D convolutional filter (*cf*_1_) are fused by Equation (3), which can ensure that they directly affect each other and directly affect the final output of the spatial branch separately and simultaneously.

(3) $$X_{l+1}=f(X_{l}+cf_{2}(X_{l})+cf_{1}(cf_{2}(X_{l})))$$

**Figure 2:**
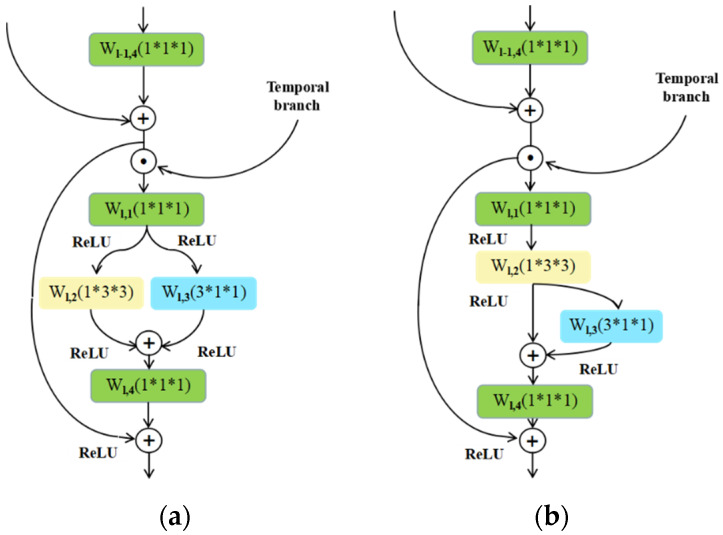
The different structures of the spatial branch developed for the STINP: (**a**) is the spatial branch in STINP-1, and (**b**) is the spatial branch in STINP-2. The yellow blocks represent the 2D convolutional filter, and the blue blocks represent the 1D convolutional filter.

In Equations (2) and (3), $X_{l}$ and $X_{l+1}$ are the input and output data of the l-th residual unit, and f denotes the *ReLU*. The detailed structures of the spatial branches in STINP-1 and STINP-2 are shown in Figure 2a,b, respectively.

From Equations (2) and (3), we find that the 2D filter and 1D filter influence the output of each layer of the spatial branch regardless of whether they affect each other. Therefore, regardless of which combination approach we take, we can evidently and directly use the appearance features from single frames and interaction features among several frames in the process of network learning, which is beneficial for improving the performance of action recognition.

Although our spatial branch is proposed by referring to Ref. [7], our STINP has two obvious differences compared with the P3D ResNets [7]. First, the pipeline of the P3D ResNets consists of only one branch, but the proposed STINP includes two branches (spatial branch and temporal branch). Second, when comparing our spatial branch developed based on P3D blocks with the P3D ResNets, the inputs of the P3D ResNets and our spatial branch are not the same. Specifically, the P3D ResNets learn the features from the RGB frames, while our proposed spatial branch learns the features from the motion feature maps scaled RGB frames (shown by ⊙ in Figure 1 and Figure 2).

### 3.3. Temporal Branch

**Figure 3:**
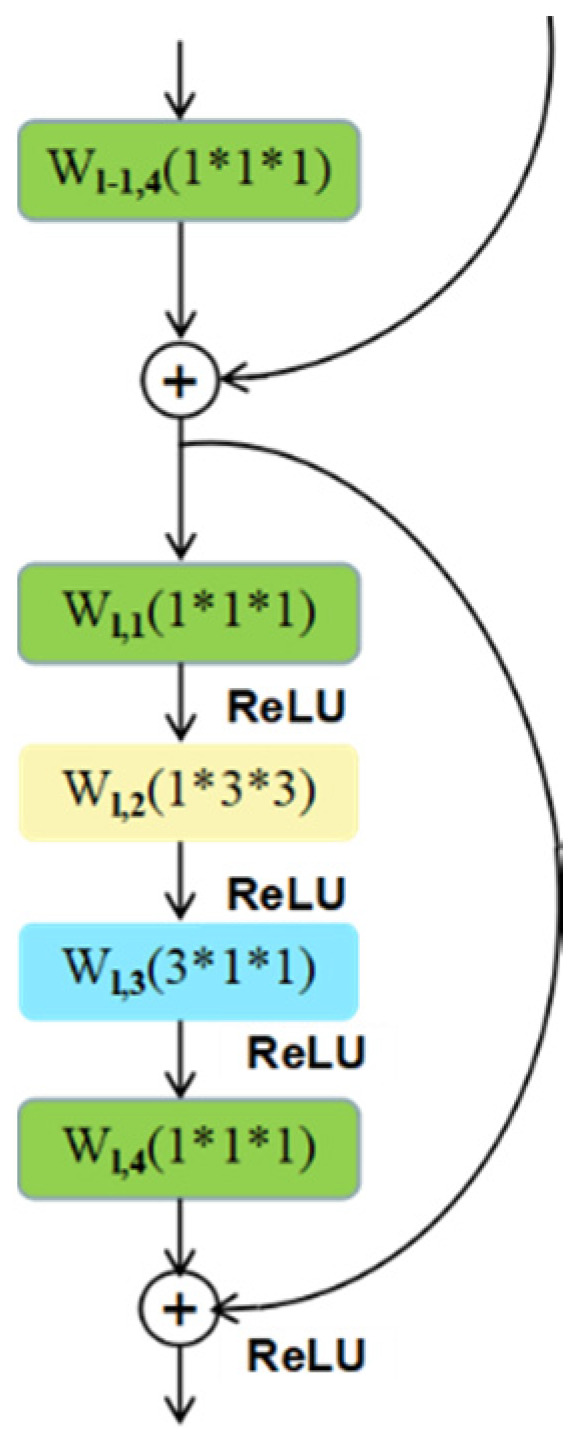
The structure of the temporal branch of the STINP. The yellow block denotes the 2D spatial convolutional filter, and the blue block represents the 1D temporal convolutional filter.

Motion features in the video can provide crucial information for action recognition and can be represented by extracting dynamic changes among the continuous frames. Optical flow [17] is a classical and effective motion representation method in the field of video processing. Therefore, we employ the precomputed optical flow images as the input of the temporal branch of the STINP. Specifically, we cascade a 2D spatial convolutional filter (*cf*_2_) and 1D temporal convolutional filter (*cf*_1_) in the “bottleneck” building block of the ResNets to learn the abstract immediate motion information from the single optical flow image and fuse them to capture long-time motion information from the continuous optical flow images. The temporal branch is shown in Figure 3 and can be expressed as:

(4) $$X_{l+1}=f(X_{l}+cf_{1}(cf_{2}(X_{l})))$$

where $X_{l}$ and $X_{l+1}$ are the input and output data of the l-th residual unit, and f denotes the *ReLU*.

### 3.4. Combination of the Spatial and Temporal Branches

To effectively and simultaneously learn the appearance representation from single frames, interaction features among several frames, and the motion representation from the optical flow images, we integrate the spatial branch and temporal branch into the STINP.

**Figure 4:**
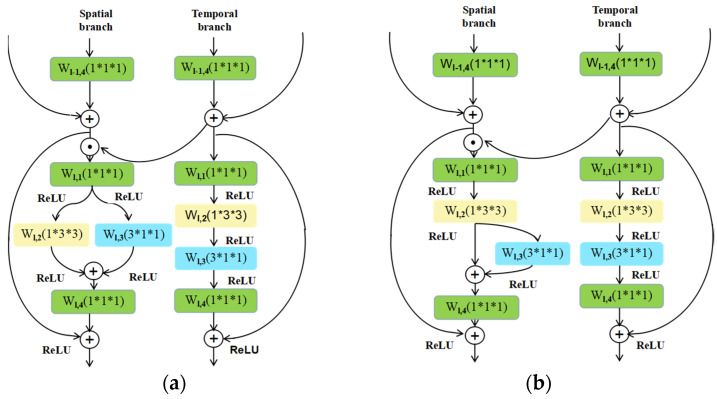
The structure of the proposed STINP. (**a**) STINP-1 and (**b**) STINP-2.

Many integration approaches have been proposed, such as fusing softmax layers [47] and max pooling operations on the output feature maps of each branch [19]. These approaches cannot fully gain the spatiotemporal features because of the late fusion. Hence, we utilize multiplication to fuse the spatial branch and temporal branch, as shown in Figure 4. Specifically, the output of the last residual unit in the temporal branch is used to multiply the input of the current residual unit in the spatial branch. The motion feature maps are used to weight the appearance feature maps pixel by pixel. The advantage of the multiplication fusion operator is twofold: (1) a multiplication fusion operator can make the spatial and temporal branches interact with each other in each residual unit during the process of the network learning, which avoids the drawback of late fusion; (2) and a multiplication fusion operator can use the motion feature to weight the appearance feature to prevent the appearance representation learning from dominating the network learning, which is beneficial to action recognition because the motion information is generally more discriminative for categorizing action [19].

## 4. Experiments

### 4.1. Datasets

To verify the performance of the proposed STINP, we evaluated it on two classical action recognition datasets: UCF101 [48] and HMDB51 [49].

**Figure 5:**
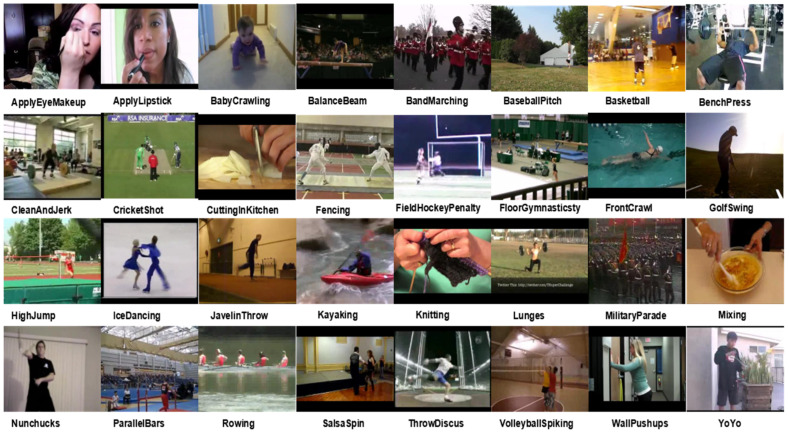
Examples of videos from the UCF101 dataset.

The UCF101 dataset consists of 13,320 real action videos across 101 action categories collected from YouTube. UCF101 is a challenging action recognition dataset due to the diversity of motions and postures within the same action class, and the significant changes in camera movement, object appearance, viewpoint, background, lighting conditions, etc. Figure 5 shows some videos from the UCF101 dataset.

**Figure 6:**
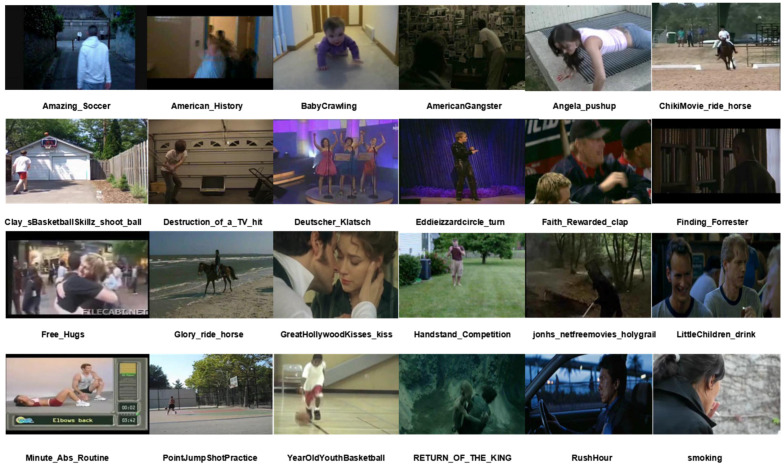
Examples of videos from the HMBD51 dataset.

The HMDB51 dataset contains 6849 videos divided into 51 action categories, and each category contains at least 101 videos. Most videos in the HMDB51 dataset are collected from movie clips, while others are from the Prelinger Archive, YouTube, Google, etc. Recognizing the action categories in HMDB51 is also difficult because of the variation in lighting conditions, backgrounds, camera types, and observation points in this dataset. Figure 6 shows some videos from the HMDB51 dataset.

### 4.2. Experimental Setup

During the experiments, we employed the ResNets [12] model pretrained on the ImageNet [50] dataset to construct our proposed STINP. Specifically, we modified the residual unit of the original ResNets to build the new blocks in the spatial branch and temporal branch, and then combined these two branches into STINP. The detailed architecture of the convolutional blocks in our proposed STINP is shown in Table 1, which should be viewed from top to bottom, left to right. The different rows represent the different layers of STINP, the brackets represent the modified residual unit adopted in our STINP, (⊙) denotes the multiplicative operation used for combining the spatial and temporal branches, (ℑ) denotes the STINP-1 architecture and (ℜ) denotes the STINP-2 architecture. The numbers in the brackets, e.g., (3 × 3 × 1, 64), denote the width of the filters, the height of filters, the number of filters in one group, and the number of feature maps, respectively. Each convolution block is followed by batch normalization [46] and a *ReLU* [37]. From Table 1, we can see that the first layer in our proposed STINP is a 7 × 7 × 1 convolution block, the second layer is a 3 × 3 × 1 maximum pooling operation, the last layer is a 7 × 7 × 1 average pooling operation, and the layers between the second layer and the last layer are several convolution blocks.

In the process of STINP training, the learning rate is initialized as 10^−2^, and it decreases twice after the verification error reaches the saturation point. To optimize the STINP, we select stochastic gradient descent (SGD) as the optimization function since we found that SGD can achieve an excellent performance compared to several other optimization functions in the experiment. In SGD, the momentum is set as 0.9. It should be noted that the input and parameter settings of the spatial branch and temporal branch are different, so we will introduce them separately in the following.

For the spatial branch, the RGB frames of the video are resized at 224 × 224 and utilized as input. The random dithering operation is adopted as a data augmentation method to increase the diversity of the RGB frames. The batch size is set as 256.

For the temporal branch, we precompute the optical flow fields for each frame and save them into images as the input of the temporal branch. When computing the optical flow, we first extract horizontal and vertical flow from 10 neighboring frames of the current frame, and then represent each frame in the video by stacking 10 optical flow fields. To enhance the generalization capabilities of the network model, the data augmentation operation, which is the same as Ref. [51], is adopted in our experiments. The images produced by augmentation are resized to 224 × 224 before they are input to the networks. To prevent overfitting, a dropout rate of 0.8 is added after the final classification layer. Considering the memory limitations of the GPU, we set the batch size as 64 in the temporal branch.

We used MATLAB to implement the proposed STINP on a computer with an NVidia GTX 1080 GPU (with GPU memory of 8 GB) and the speed of training was about 50 frames per second.

### 4.3. Experimental Results and Analysis

In all experiments, we used three training/testing splits provided separately by the official organizations of the UCF101 and HMDB51 datasets to test the proposed approach and report the average recognition accuracy of the approach on each dataset.

#### 4.3.1. Analyzing the Performances of STINP-1 and STINP-2

As described in Section 3, we proposed two different structures of spatial branches by combining 1D and 2D convolutional filters in different ways. Hence, the proposed STINP has two different architectures, STINP-1 and STINP-2, as shown in Figure 4. To evaluate the performances of these two versions of STINP, we compared them using the UCF101 and HMDB51 datasets, and we also evaluated the recognition accuracies of each branch in STINP. In addition, it should be noted that we adopted ResNets with different depths (ResNets-50 and ResNets-152) to perform this experiment, in order to determine the effect of the depth of the network on the performance of the STINP; thus, we were able to select an optimal setting for STINP. The experimental results are summarized in Table 2, Table 3, Table 4 and Table 5.

From Table 2, Table 3, Table 4 and Table 5, the following points can be observed. (1) Both STINP-1 and STINP-2 can achieve good performance for action recognition in videos, but STINP-1 generally outperforms STINP-2. To be specific, on the UCF101 dataset, all results of STINP-1 are better than the results of STINP-2; on the HMDB51 dataset, the results of STINP-1 and STINP-2 show a tie. This means both proposed structures of the spatial branch in STINP are meaningful, but combining the 1D convolutional filter and 2D convolutional filter parallelly in the spatial branch is slightly better. (2) Fusing the spatial and temporal branches can greatly improve the action recognition accuracies compared to the models adopting only one branch. For example, in Table 3, the spatial branch and the temporal branch of STINP-1 achieve 89.8% and 86.4% recognition rates, respectively, on the UCF101 dataset, but when using a multiplication operation to fuse the two branches, the accuracy increases to 94.4%. This is because combining the spatial and temporal branches can effectively capture the appearance feature, the relationship cues between the adjacent frames, and the motion feature in videos, which is beneficial for the action recognition. 3) When adopting ResNets-50 for the spatial branch and ResNets-152 for the temporal branch, STINP-1 and STINP-2 can obtain the best recognition accuracies on the two datasets; this may be because ResNets-152 in the spatial branch will lead to model overfitting.

To further compare the performances of the STINP-1 and STINP-2, in Table 6, we provide the top-5 recognition accuracies when adopting the best setting (ResNets-50 for the spatial branch and ResNets-152 for the temporal branch). From Table 6, we find that the performance of STINP-1 is still superior to STINP-2, which is consistent with the conclusions obtained from Table 2, Table 3, Table 4 and Table 5.

In summary, constructing two branches for separately learning the spatial and temporal information is reasonable, so that both STINP-1 and STINP-2 can achieve good performance for action recognition in video. When dealing with the practical tasks, we recommend the users first choose STINP-1 because STINP-1 generally outperforms STINP-2 in our experiments. However, if the dataset is particularly challenging, such as HMDB51 (HMDB51 is a more challenging dataset because it has more complex backgrounds and context environments [52]), the users also can select STINP-2, because STINP-1 is not always better than STINP-2 on the HMDB51 dataset.

#### 4.3.2. Comparing STINP with the State-of-the-Art

To verify the validity of the proposed STINP, we compared it with several current state-of-the-art video action recognition methods. Because Table 2, Table 3, Table 4, Table 5 and Table 6 demonstrate that the performance of STINP-1 is slightly better than STINP-2, we only compare STINP-1, which uses ResNets-50 for the spatial branch and ResNets-152 for the temporal branch, with other compared methods to avoid tautology. Our STINP is inspired by Refs. [7,19]; when comparing the STINP with these studies, there is one point that should be mentioned: we compare the STINP with the methods without improved dense trajectory (IDT; [53]) in Refs. [7,19], since IDT is a hand-crafted feature and can also be combined with our STINP. We thus hope to ignore the influence of the IDT to mainly focus on comparing the effectiveness of the frameworks of our STINP and Refs. [7,19].

Table 7 gives the comparison results (average top-1 accuracy). From Table 7, we can see that the proposed STINP can acquire better performance than other comparable approaches (for fairness we do not include models pre-trained on the Kinetics [43] dataset), including the models proposed in Refs. [7,19]. Specifically: (1) our proposed STINP yields a better performance than the P3D ResNets [7]; this is because the architecture of the P3D ResNets consists of only one branch, meaning that it cannot fully capture the motion information in the video, while the proposed STINP includes two branches (spatial branch and temporal branch) so that it can effectively learn both the motion and appearance features of the video. (2) Our proposed STINP is superior to Spatiotemporal Multiplier ConvNet [19]; this is because the spatial branch in Spatiotemporal Multiplier ConvNet can acquire the appearance features of the single frames, while the spatial branch in our STINP can not only learn the appearance features from the individual frames but also the interactive cues among the neighboring frames.

To summarize, the proposed STINP achieves the highest recognition accuracies in the experiments, e.g., 94.4% on the UCF101 dataset and 69.6% on the HMDB51 dataset. This is because the STINP introduces the pseudo3D structure into the residual units of ResNets to build the spatial branch and combines the spatial branch and temporal branch by the multiplication operation. That is, we fuse the two-stream and pseudo3D architecture into a unified framework. Hence, the proposed STINP can ensure that (1) the STINP can not only extract the appearance feature and the motion cues in the video but also simultaneously capture the relationship information of contiguous frames; (2) the spatial and temporal information can influence each other during the learning process of the STINP by a multiplication operation, which avoids the drawback of a two-stream network, e.g., it does not truly learn the spatiotemporal information of the video because it only lately fuses the outputs of the softmax layer.

## 5. Conclusions

This paper proposed a novel and effective action recognition method, named the spatiotemporal interaction residual network with pseudo3D (STINP), which possesses the advantages of 3D and two-stream network architectures since it introduces the pseudo3D block into the network architecture with two branches. In particular, each branch in our proposed STINP is constructed based on ResNets, and two branches are combined by a multiplication operation. Hence, the proposed STINP can simultaneously learn the appearance, the interactive and complementary information of several continuous frames, and the dynamic motion information of the video. The performance of STINP is verified on the classical action recognition datasets, namely, UCF101 and HMDB51. In the future, we will investigate pre-training the proposed STINP on a large-scale action recognition dataset, Kinetics [43], to further improve the action recognition accuracies, and combine our work with the Optical Flow Guided Feature (OFF) [67] and IDT [53] to further improve the performance of STINP.

## Figures and Tables

**Table 1:** The detailed architecture of convolutional blocks in our proposed STINP.

Layer Name	Blocks
conv1	7 × 7 × 1,64
pool1	3 × 3 × 1 max stride 2
conv2_i	[1×1×1,643×3×1,641×1×1,256] ⊙[1×1×1,641×3×3,643×1×1,64 (ℑ/ℜ)1×1×1,256][1×1×1,643×3×1,641×1×1,256]
conv3_i	[1×1×1,1283×3×1,1281×1×1,512] ⊙[1×1×1,1281×3×3,1283×1×1,128 (ℑ/ℜ)1×1×1,512][1×1×1,1283×3×1,1281×1×1,512]
conv4_i	[1×1×1,2563×3×1,2561×1×1,1024] ⊙[1×1×1,2561×3×3,2563×1×1,256 (ℑ/ℜ)1×1×1,1024][1×1×1,2563×3×1,2561×1×1,1024]
conv5_i	[1×1×1,5123×3×1,5121×1×1,2048] ⊙[1×1×1,5121×3×3,5123×1×1,512 (ℑ/ℜ)1×1×1,2048][1×1×1,5123×3×1,5121×1×1,2048]
Pool5	7 × 7 × 1 average

**Table 2:** Comparison results (Top-1) of STINP-1 and STINP-2 using ResNets-50 for the spatial branch and ResNets-50 for the temporal branch.

Model	Branch	UCF101	HMDB51
STINP-1	Spatial branch-1	84.00%	53.20%
	Temporal branch	86.00%	62.10%
	Fusion	93.40%	66.70%
STINP-2	Spatial branch-2	83.20%	53.00%
	Temporal branch	86.00%	62.10%
	Fusion	93.00%	67.10%

**Table 3:** Comparison results (Top-1) of STINP-1 and STINP-2 using ResNets-50 for the spatial branch and ResNets-152 for the temporal branch.

Model	Branch	UCF101	HMDB51
STINP-1	Spatial branch-1	89.80%	61.60%
	Temporal branch	86.40%	60.80%
	Fusion	94.40%	69.60%
STINP-2	Spatial branch-2	87.50%	59.00%
	Temporal branch	86.60%	60.20%
	Fusion	94.00%	69.00%

**Table 4:** Comparison results (Top-1) of STINP-1 and STINP-2 using ResNets-152 for the spatial branch and ResNets-50 for the temporal branch.

Model	Branch	UCF101	HMDB51
STINP-1	Spatial branch-1	86.30%	54.10%
	Temporal branch	85.00%	61.80%
	Fusion	93.60%	68.70%
STINP-2	Spatial branch-2	85.80%	53.80%
	Temporal branch	85.00%	61.20%
	Fusion	93.50%	68.50%

**Table 5:** Comparison results (Top-1) of STINP-1 and STINP-2 using ResNets-152 for the spatial branch and ResNets-152 for the temporal branch.

Model	Branch	UCF101	HMDB51
STINP-1	Spatial branch-1	85.80%	56.60%
	Temporal branch	86.10%	60.00%
	Fusion	93.70%	67.80%
STINP-2	Spatial branch-2	86.20%	55.80%
	Temporal branch	84.50%	58.80%
	Fusion	93.30%	68.00%

**Table 6:** Comparison results (Top-5) of STINP-1 and STINP-2 using ResNets-50 for the spatial branch and ResNets-152 for the temporal branch.

Model	UCF101	HMDB51
STINP-1	99.50%	91.60%
STINP-2	98.80%	91.00%

**Table 7:** Comparison of the proposed STINP and the other methods.

Methods	UCF101	HMDB51
IDT [53]	86.40%	61.70%
Spatiotemporal ConvNet [8]	65.40%	—
Long-term recurrent ConvNet [54]	82.90%	—
Composite LSTM Model [55]	84.30%	44.00%
Two-Stream ConvNet [17]	88.00%	59.40%
P3D ResNets (Without IDT) [7]	88.60%	—
Two-Stream+LSTM [56]	88.60%	—
C3D [42]	85.20%	-
Res3D [57]	85.80%	54.90%
Dynamic Image Networks [58]	76.90%	42.80%
Dynamic Image Networks + IDT [58]	89.10%	65.20%
Asymmetric 3D-CNN (RGB+RGBF+IDT) [59]	92.60%	65.40%
T3D [60]	93.20%	63.50%
TDD+IDT [61]	91.50%	65.90%
Conv Fusion (Without IDT) [47]	92.50%	65.40%
Transformations [51]	92.40%	62.00%
VideoLSTM + IDT [62]	92.20%	64.90%
Hierarchical Attention Networks [63]	92.70%	64.30%
Spatiotemporal Multiplier ConvNet [19]	94.20%	68.90%
Sequential Learning Framework [64]	90.90%	65.70%
T-ResNets (Without IDT) [16]	93.90%	67.20%
TSN (2 modalities) [65]	94.00%	68.50%
Spatiotemporal Heterogeneous Two-stream Network [66]	94.40%	67.20%
Our proposed STINP	94.40%	69.60%

IDT is the abbreviation of Improved Dense Trajectory; ConvNet is the abbreviation of Convolutional Network; LSTM is the abbreviation of Long Short-Term Memory; P3D ResNets is the abbreviation of Pseudo-3D Residual Networks; C3D is the abbreviation of Convolutional 3D; Res3D is the abbreviation of 3D Residual Convolutional Network; 3D-CNNs is the abbreviation of 3D Convolutional Neural Networks; T3D is the abbreviation of Temporal 3D Convolutional Network; TDD is the abbreviation of Trajectory-pooled Deep-convolutional Descriptors; Conv Fusion is the abbreviation of Convolutional Two-Stream Network Fusion; T-ResNets is the abbreviation of Temporal Residual Networks; TSN is the abbreviation of Temporal Segment Networks.
